# Proteomic response to phosphorus deficiency and aluminum stress of three aluminum-tolerant phosphobacteria isolated from acidic soils

**DOI:** 10.1016/j.isci.2023.107910

**Published:** 2023-09-14

**Authors:** Patricio Javier Barra, Paola Duran, Mabel Delgado, Sharon Viscardi, Stéphane Claverol, Giovanni Larama, Marc Dumont, María de la Luz Mora

**Affiliations:** 1 Center of Plant, Soil Interaction and Natural Resources Biotechnology, Scientific and Technological Bioresource Nucleus, Universidad de La Frontera, Temuco 4811230, Chile; 2 Biocontrol Research Laboratory, Universidad de La Frontera, Temuco 4811230, Chile; 3 Facultad de Ciencias Agropecuarias y Medioambiente, Departamento de Producción Agropecuaria, Universidad de La Frontera, Temuco 4811230, Chile; 4 Escuela de la Salud, Campus San Francisco, Universidad Católica de Temuco, Temuco 4811230, Chile; 5 Plateforme Protéome, Centre Génomique Fonctionnelle de Bordeaux, Université de Bordeaux, Bordeaux, France; 6 School of Biological Sciences, University of Southampton, Southampton, UK

**Keywords:** soil science, Microbiology, Bacteriology, proteomics

## Abstract

Aluminum (Al)-tolerant phosphobacteria enhance plant growth in acidic soils by improving Al complexing and phosphorus (P) availability. However, the impact of Al stress and P deficiency on bacterial biochemistry and physiology remains unclear. We investigated the single and mutual effects of Al stress (10 mM) and P deficiency (0.05 mM) on the proteome of three aluminum-tolerant phosphobacteria: *Enterobacter* sp. 198, *Enterobacter* sp. RJAL6, and *Klebsiella* sp. RCJ4. Cultivated under varying conditions, P deficiency upregulated P metabolism proteins while Al exposure downregulated iron-sulfur and heme-containing proteins and upregulated iron acquisition proteins. This demonstrated that Al influence on iron homeostasis and bacterial central metabolism. This study offers crucial insights into bacterial behavior in acidic soils, benefiting the development of bioinoculants for crops facing Al toxicity and P deficiency. This investigation marks the first proteomic study on the interaction between high Al and P deficiency in acid soils-adapted bacteria.

## Highlights

• Al stress and P deficit effects on the proteome of Al-tolerant bacteria were assessed
• Al stress triggered changes in Fe homeostasis and central metabolism in bacteria
• P deficit upregulated organophosphorus compound uptake proteins in bacteria

## Graphical abstract

**Figure:**
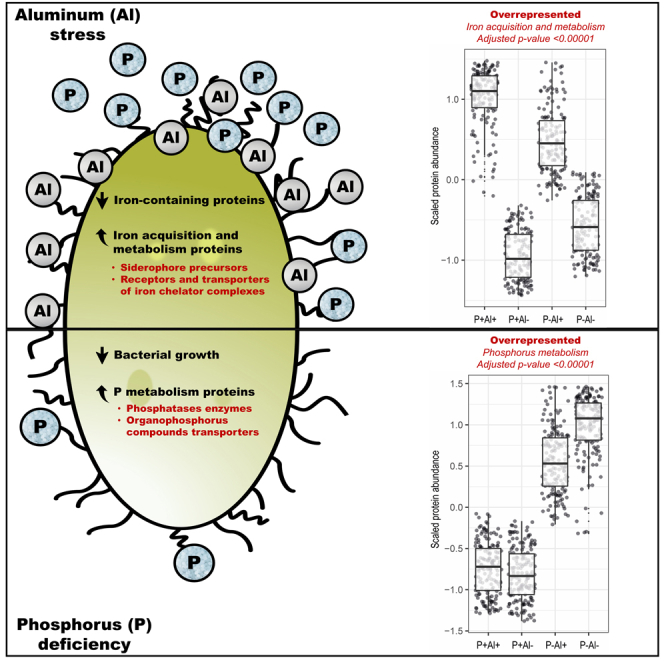

## Introduction

Although aluminum (Al) is the most abundant metallic element in soils, at near-neutral pH and above it occurs predominantly integrated into insoluble and biologically innocuous forms, such as aluminosilicates and precipitates.^1^^,^^2^^,^^3^ In acidic soil, Al is solubilized to its ionic form, Al^3+^, which is toxic to organisms including many species of plants^3^ and bacteria.^4^^,^^5^^,^^6^ Although not fully elucidated, there is evidence that Al exerts toxicity by perturbing the homeostasis of several cations fundamental for the normal cell function, including calcium (Ca), magnesium (Mg) and, in particular, iron (Fe).^4^^,^^7^^,^^8^ Aluminum can compete for Fe-binding sites in bacteria^9^^,^^10^^,^^11^ and human cells.^12^^,^^13^^,^^14^ Aluminum can also exert a pro-oxidant activity through promoting the formation of Al-superoxide radical complexes able to reduce Fe^3+^ to Fe^2+^ via the Fenton reaction, leading to the generation of reactive oxygen species (ROS) that lead to cell damage.^4^^,^^7^^,^^8^^,^^15^ This process could possibly be favored by the accumulation of intracellular free Fe as a consequence of its competition with Al.^4^^,^^8^

In acidic soils, naturally occurring toxic levels of Al usually coincide with scarce bioavailability of phosphorus (P) as a result of orthophosphate anions (Pi), the labile form of P assimilable by plants, forming inorganic complexes with Al and Fe oxides.^16^^,^^17^ In addition, 30–60% of total soil P is integrated into complex organic molecules (soil organic P; Po),^18^ which are not immediately bioavailable to plants. As a primary constituent of vital biomolecules and a key constituent of cellular processes, P is an indispensable macronutrient for all levels of life. The availability of Pi is thus a primary factor that governs the survival, success, and performance of the plants and microorganisms living in acidic soils.^19^^,^^20^ Therefore, both Al toxicity and Pi deficiency are two major factors restricting crop productivity in acidic soils.^21^^,^^22^

Naturally occurring biota have developed strategies to overcome the hostile conditions in acidic soil. For example, the secretion of Al-chelating organic compounds by plant roots is a well-documented mechanism of Al tolerance, which together with the action of phosphatase enzymes enhances Pi availability in the rhizosphere.^23^^,^^24^^,^^25^ Plants also have an active role in attracting beneficial microorganisms from surrounding soil and, therefore, in assembling its rhizosphere microbiome.^26^^,^^27^ Plant-associated bacteria are known for the ability to improve the performance of their host under stressful conditions by several well-documented mechanisms, such as the production and release of phytohormones, siderophores and the activity of the enzyme 1-aminocyclopropane-1-carboxylate (ACC) deaminase, probably coupled to other still unknown mechanisms.^28^^,^^29^ An important group of plant-associated bacteria, referred to as phosphobacteria, contribute to enhancing P availability in the rhizosphere by releasing low-molecular-weight organic acids and phosphatase enzymes that directly influence the mineralization and solubilization of inorganic and organic P compounds, respectively.^30^^,^^31^^,^^32^ This bacterial function is fundamental in several types of soils, such as andisols, which often have high concentrations of highly recalcitrant organic compounds.^2^

Phosphobacteria have an important ecological role promoting plant growth and health in acidic soils.^33^ However, bacterial responses to high Al levels, particularly under P deficiency, are poorly studied and information at the molecular level, such as protein expression, is practically non-existent. Only a few studies have suggested several physiological mechanisms of Al tolerance in bacteria including adjustment in the activity of pivotal enzymes in the tricarboxylic acid (TCA) cycle, and the production and release of metal sequestering and chelating compounds, such as low-molecular-weight organic acids, phosphatidylethanolamine, and siderophores.^4^^,^^30^^,^^34^^,^^35^^,^^36^^,^^37^ For example, Mora et al., 2017^37^ determined that five Al-tolerant phosphobacteria strains able to tolerate over 10 mM Al, a concentration that is usually toxic to soil bacteria,^38^ could alleviate Al stress by forming extracellular Al^3+^–siderophore complexes. These bacterial strains identified as *Klebsiella* sp. RC3, *Stenotrophomonas* sp. RC5, *Klebsiella* sp. RCJ4, *Serratia* sp. RCJ6, and *Enterobacter* sp. RJAL6 also adjusted their patterns of organic acid release under high Al levels, including the relative expression of the genes encoding the enzyme malate dehydrogenase (*mdh*).^30^ In contrast, the physiological and molecular mechanisms of response to P starvation in soil bacteria have been much better studied, and it is well known that the activation of the phosphate (Pho) regulon plays a central role.^39^ The most common group of proteins reported to be induced by the Pho regulon in response to P starvation include (i) extracellular P scavenger enzymes responsible for obtaining Pi from organic compounds; (ii) transporters of different P forms; and (iii) proteins involved in the intracellular storage and cycling of P.^39^^,^^40^ The induction of alkaline phosphatase and transporters of organic and inorganic phosphate in response to P deficiency have been widely described, but there is evidence of many more poorly studied or uncharacterized processes that respond to P deficiency.^20^^,^^40^ For example, Lidbury et al., (2016, 2021) showed in genomic and proteomic studies that several additional proteins are overrepresented in bacteria in response to P deficiency, including putative nucleases, phosphotriesterases, putative phosphonate transporters, and outer membrane proteins.

Therefore, considering the gaps in knowledge in the behavior of plant-associated bacteria naturally adapted to acidic soils, the objective of this study was to evaluate by label-free quantitative proteomics the response of three Al-tolerant phosphobacteria strains to Al stress, P deficiency, and their combination. The information obtained in this study provides the basis to understand the molecular responses of plant-associated bacteria to the environmental stresses typically found in acidic soils i.e., Al stress and P deficiency, which is crucial to understand the adaptation processes of microorganisms to acidic soils, knowledge that in turn could be used in the formulation of efficient biofertilizers.

## Results

### Overall bacterial response to P and Al

We evaluated the effect of P deficiency and Al stress on three Al-tolerant phosphobacteria strains by comparing their proteomes under P-deplete (0.05 mM) or P-replete (1.4 mM) conditions in both the presence (10 mM) and in the absence (0 mM) of Al. In general, the bacteria had a behavior similar to that described by Barra et al., 2018.^30^ That is, the exponential growth phase was not significantly (p value > 0.05) affected by Al (OD_600_: 0.42–0.54) when grown under P-replete conditions (P + Al+) as compared with the control (P + Al–; OD_600_: 0.52–0.60). These densities were reached after 20–26 h of culture. In contrast, under P deficiency, both in the presence (P–Al+; OD_600_: 0.26–0.36) and absence (P–Al–; OD_600_: 0.28–0.38) of Al, the exponential growth phase was significantly (p ≤ 0.05) lower as compared with the control (P + Al–). These densities were reached after 20–32 h of culture. It is worth noting that, except for the strain *Klebsiella* sp. RCJ4, Al did not induce a significant change in bacterial growth under P deficiency either (Table S2).

Based on the recognition of at least two unique peptides, a total of 2,176, 2,005, and 2,202 proteins were identified and quantified in the bacterial strains 198, RJAl6 and RCJ4, respectively (Table 1; Table S3), of which 16.2%, 21.8%, and 25.9% showed significant differences (p value ≤ 0.05, log_2_ fold change ≥ 2) in abundance in at least one of the treatments (P + Al+, P–Al+, P–Al–) when compared with the control (P + Al–) (Table S3). These percentages also include proteins that were detected in some of the treatments but were absent in the control, or vice versa (presence/absence) (Table S4). Of the total proteins identified, 1,174 were common to the three bacterial strains, in contrast to the 293, 368, and 313 proteins that were exclusively found in the strains 198, RJAl6 and RCJ4, respectively (Table S5). The greatest variety of proteins identified in the three bacterial strains was found in the control treatment (P + Al–) with 2,159, 1,957, and 2,183 in the strains 198, RJAl6 and RCJ4, respectively (Table S3). 66–69% of the identified proteins were functionally classified into twentysix categories based on SEED functional classification (Table 1; Table S4). The remaining proteins (31–34%) were not categorized within any SEED subsystem (Table 1).

**Table 1:** Number of total proteins identified and differentially expressed in the strains *Enterobacter* sp. 198, *Enterobacter* sp. RJAL6 and *Klebsiella* sp. RCJ4 when subject to a high Al (P + Al+), P deficiency and high Al (P– Al+) and P deficiency (P– Al–)

Subsystems	Number of total identified proteins			Number of differentially expressed proteins in P + Al+						Number of differentially expressed proteins in P- Al+						Number of differentially expressed proteins in P- Al-					
	198	RJAl6	RCJ4	198		RJAl6		RCJ4		198		RJAl6		RCJ4		198		RJAl6		RCJ4	
				Up	Down	Up	Down	Up	Down	Up	Down	Up	Down	Up	Down	Up	Down	Up	Down	Up	Down
Amino Acids and Derivatives	211	214	224	0	8	7	4	0	20	0	12	7	18	4	14	0	10	9	17	3	11
Carbohydrates	247	225	346	0	9	11	11	2	31	5	12	9	13	8	25	3	10	12	13	6	19
Cell Division and Cell Cycle	29	19	30	0	3	0	0	0	6	0	2	0	1	1	1	0	6	0	1	1	2
Cell Wall and Capsule	107	100	128	0	5	0	6	0	16	0	1	1	13	0	11	0	5	1	12	0	12
Cofactors, Vitamins, Prosthetic Groups, Pigments	136	116	163	1	11	1	2	0	31	1	2	4	3	2	16	0	6	3	5	2	14
DNA Metabolism	61	47	76	0	7	0	0	0	13	0	4	1	1	0	4	0	6	1	1	0	8
Dormancy and Sporulation	2	2	2	0	0	0	0	0	1	0	0	0	1	0	0	0	0	0	1	0	0
Fatty Acids, Lipids, and Isoprenoids	53	51	60	0	3	5	3	0	10	1	2	0	4	3	4	0	5	1	5	2	3
Iron acquisition and metaboli	28	34	48	8	0	13	2	25	0	12	1	10	1	31	0	0	2	2	3	2	5
Membrane Transport	82	93	84	5	2	6	6	6	16	5	3	9	9	8	8	2	5	12	11	1	7
Metabolism of Aromatic Compounds	11	21	22	0	0	1	1	1	1	0	2	0	0	1	2	0	3	0	1	2	2
Miscellaneous	28	27	39	0	2	1	4	0	4	0	2	1	5	0	1	0	2	1	4	0	2
Motility and Chemotaxis	41	28	11	2	2	4	0	0	0	1	0	3	5	0	0	0	3	2	4	0	0
Nitrogen Metabolism	19	14	19	0	3	0	0	0	4	0	2	0	2	0	3	0	2	0	2	0	1
Nucleosides and Nucleotides	75	66	82	0	2	3	3	1	12	0	1	4	4	1	2	0	2	0	4	0	1
Phages, Prophages, Transposable elements, Plasmids	1	1	1	0	0	0	0	0	0	0	0	0	0	0	0	0	0	0	0	0	0
Phosphorus Metabolism	31	35	32	0	4	2	1	0	8	9	1	11	1	7	4	7	1	12	2	7	3
Potassium metabolism	12	14	15	0	3	0	1	0	4	0	1	0	4	0	4	0	1	0	5	0	3
Protein Metabolism	176	144	191	1	6	2	12	2	22	1	4	0	8	0	18	1	33	0	10	0	23
Regulation and Cell signaling	75	44	81	0	10	1	1	0	22	0	3	1	6	1	11	0	11	1	5	1	13
Respiration	63	63	68	0	5	0	12	0	17	0	5	0	11	0	11	0	2	0	10	0	7
RNA Metabolism	112	72	122	0	10	1	2	0	18	0	2	1	6	0	9	0	13	1	8	0	12
Secondary Metabolism	4	3	4	0	0	0	0	0	0	0	0	0	0	0	0	0	0	1	0	0	0
Stress Response	106	98	114	1	7	1	13	0	20	2	7	4	7	0	9	0	7	4	5	0	7
Sulfur Metabolism	34	25	36	0	5	0	1	0	2	0	2	0	1	0	1	0	4	0	2	1	2
Virulence	75	59	76	0	8	0	6	2	18	1	6	1	11	3	12	0	14	1	12	2	9
Total categorized proteins	1819	1615	2074	18	115	59	91	39	296	38	77	67	135	70	170	13	153	64	143	30	166
Uncategorized proteins	691	685	840	6	61	18	49	17	116	16	27	21	78	25	63	4	69	22	91	16	68
Total proteins identified	2176^a^	2005^a^	2202^a^	24	176	77	140	56	412	54	104	88	213	95	233	17	222	86	234	46	234

a Several proteins can be categorized into more than one subsystem.

**Figure 1:**
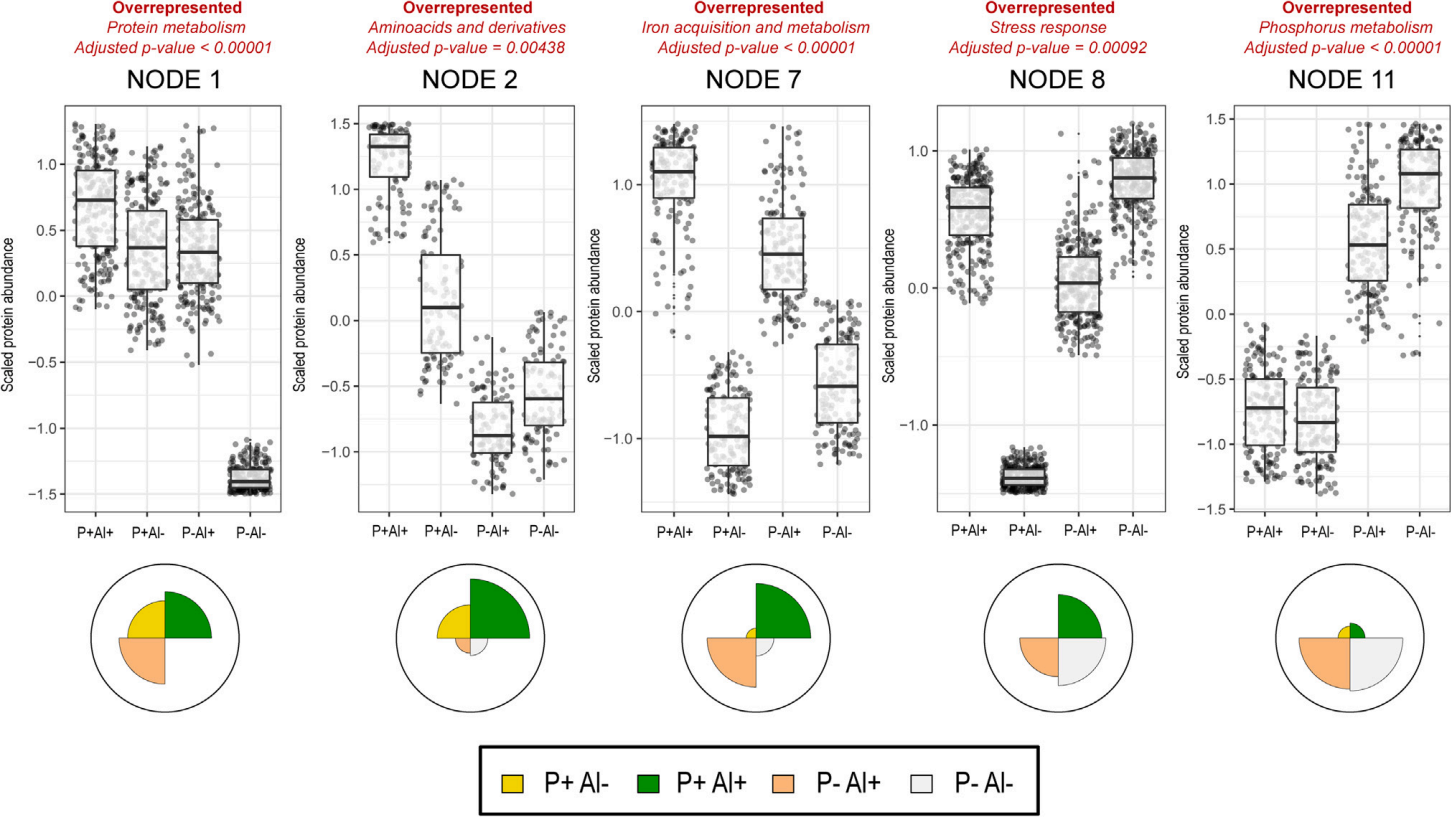
Principal Component Analysis (PCA) with Self-Organizing Map (SOM) neural network of protein expression data for the strain *Enterobacter* sp. 198 when subject to high Al (P+ Al+), P deficiency and high Al (P– Al+), P deficiency (P– Al–), and unstressed conditions (control, P+ Al–) All identified proteins in each treatment were grouped into twelve clusters (nodes) according to their relative abundance profiles. For further functional classification of each node, an enrichment analysis in terms of SEED categories (twentysix categories) was performed. The PCA-SOM analysis showed that five nodes were clustered with proteins showing significantly (p ≤ 0.005) different patterns of abundance among treatments.

**Figure 2:**
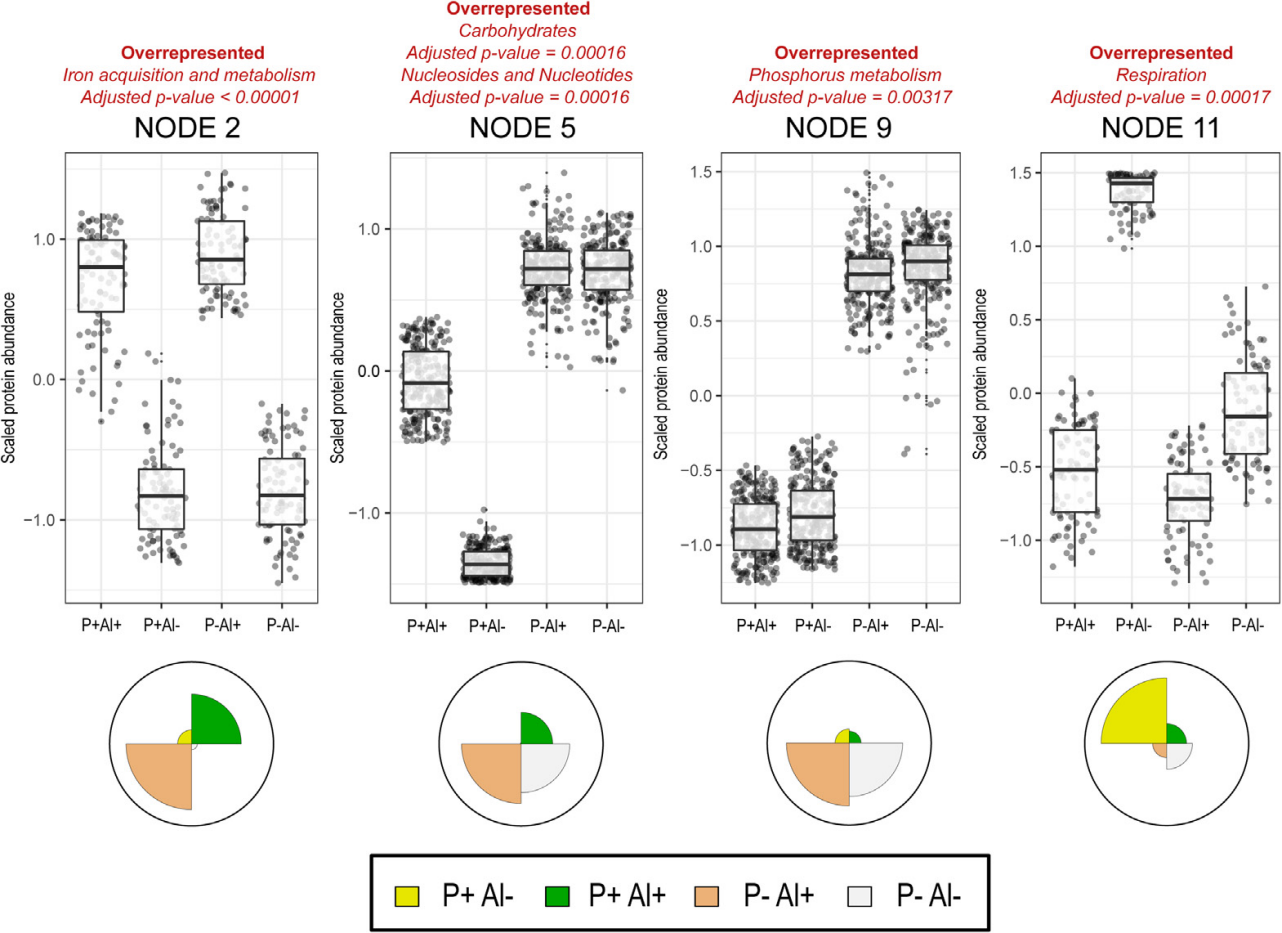
Principal Component Analysis (PCA) with Self-Organizing Map (SOM) neural network of protein expression data for the *Enterobacter* sp. RJAL6 when subject to high Al (P+ Al+), P deficiency and high Al (P– Al+), P deficiency (P– Al–), and unstressed conditions (control, P+ Al–) All identified proteins in each treatment were grouped into twelve clusters (nodes) according to their relative abundance profiles. For further functional classification of each node, an enrichment analysis in terms of SEED categories (twentysix categories) was performed. The PCA-SOM analysis showed that four nodes were clustered with proteins showing significantly (p ≤ 0.005) different patterns of abundance among treatments.

**Figure 3:**
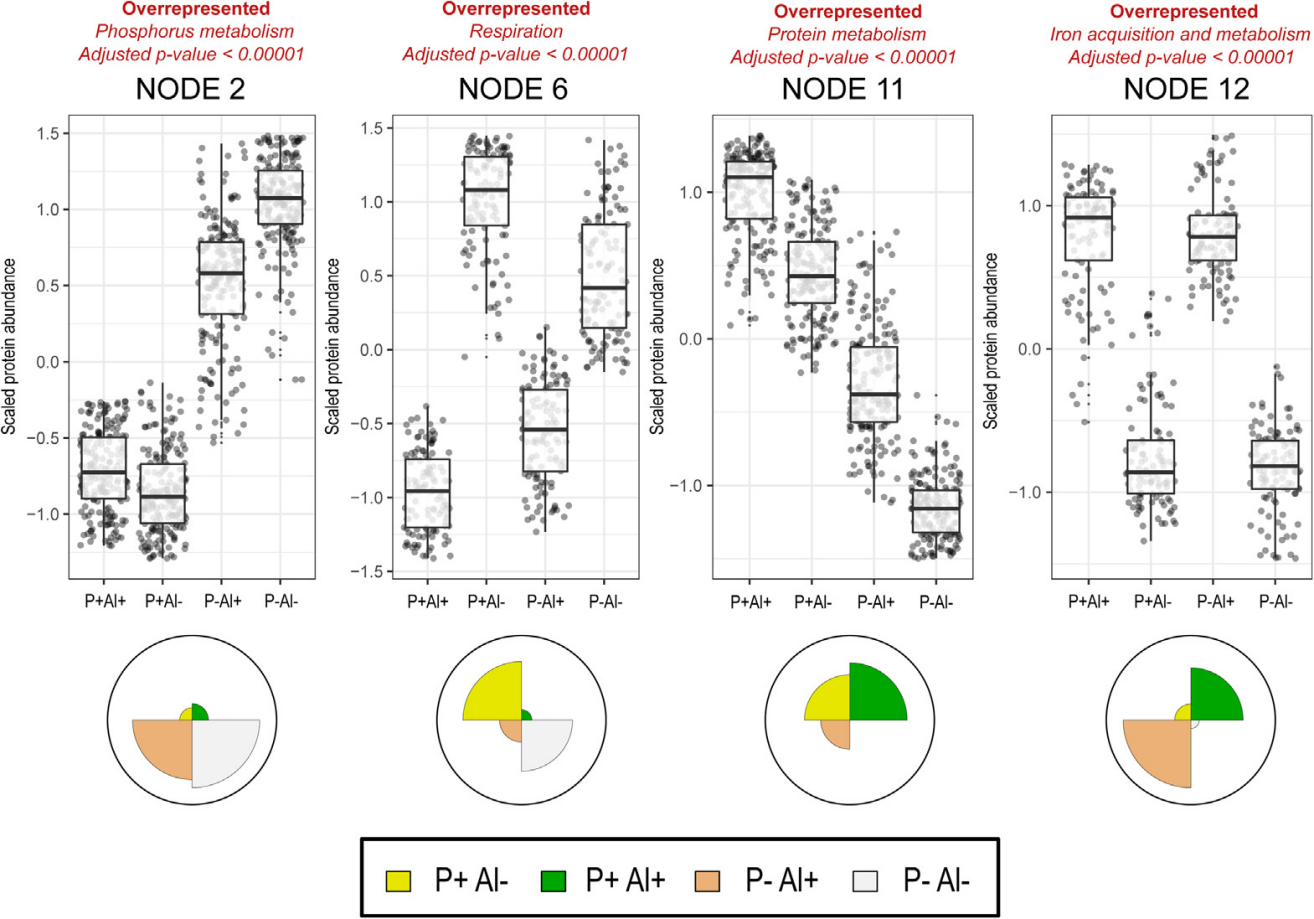
Principal Component Analysis (PCA) with Self-Organizing Map (SOM) neural network of protein expression data for the *Klebsiella* sp. RCJ4 when subject to high Al (P+ Al+), P deficiency and high Al (P– Al+), P deficiency (P– Al–), and unstressed conditions (control, P+ Al–) All identified proteins in each treatment were grouped into twelve clusters (nodes) according to their relative abundance profiles. For further functional classification of each node, an enrichment analysis in terms of SEED categories (twentysix categories) was performed. The PCA-SOM analysis showed that four nodes were clustered with proteins showing significantly (p ≤ 0.005) different patterns of abundance among treatments.

To understand and visualize the patterns of protein abundance under the conditions of high Al, P deficiency and the combination of both factors, a PCA-SOM clustering analysis was performed. For each bacterial strain, all identified proteins were grouped into twelve clusters (nodes) according to their relative abundance profiles. For further functional classification of each node, an enrichment analysis was performed in terms of SEED categories (twentysix categories). The PCA-SOM analysis showed that five nodes in strain 198 (Figure 1) and four nodes in strains RJAl6 and RCJ4 (Figures 2 and 3, respectively) were clustered with proteins showing significantly (p ≤ 0.005) different patterns of abundance among treatments. Only two SEED categories, “iron acquisition and metabolism” and “phosphorus metabolism,” were consistently found in the three bacterial strains showing significant differences in any of treatments with respect to the control. Several additional SEED categories of proteins were also significantly (p ≤ 0.05) affected by the treatments, however, the expression patterns and categories affected were strain specific.

### Bacterial response to high Al (P+ Al+)

The effect of Al on the proteome of three Al-tolerant bacterial strains was evaluated in the P + Al+ medium and the results were contrasted against the control medium (P + Al–). When the bacterial strains 198, RJAl6 and RCJ4 were grown in the presence of Al, 1,872, 1,882, and 1,883 proteins were identified, respectively (Table S3). Comparison of bacteria grown in the presence of Al versus the control showed a total of 179, 179, and 421 proteins with significant differences in abundance (Table S3; p value ≤ 0.05, log_2_ fold-change ≥ 2). Among the significantly expressed proteins, most (87.0%, 65.4%, and 88.1% in the strains 198, RJAl6 and RCJ4, respectively) were down-regulated or completely inhibited with Al (Table S4). Only 13.0%, 34.6%, and 11.9% of the proteins were induced by Al in the strains 198, RJAl6 and RCJ4, respectively (Table S4).

**Figure 4:**
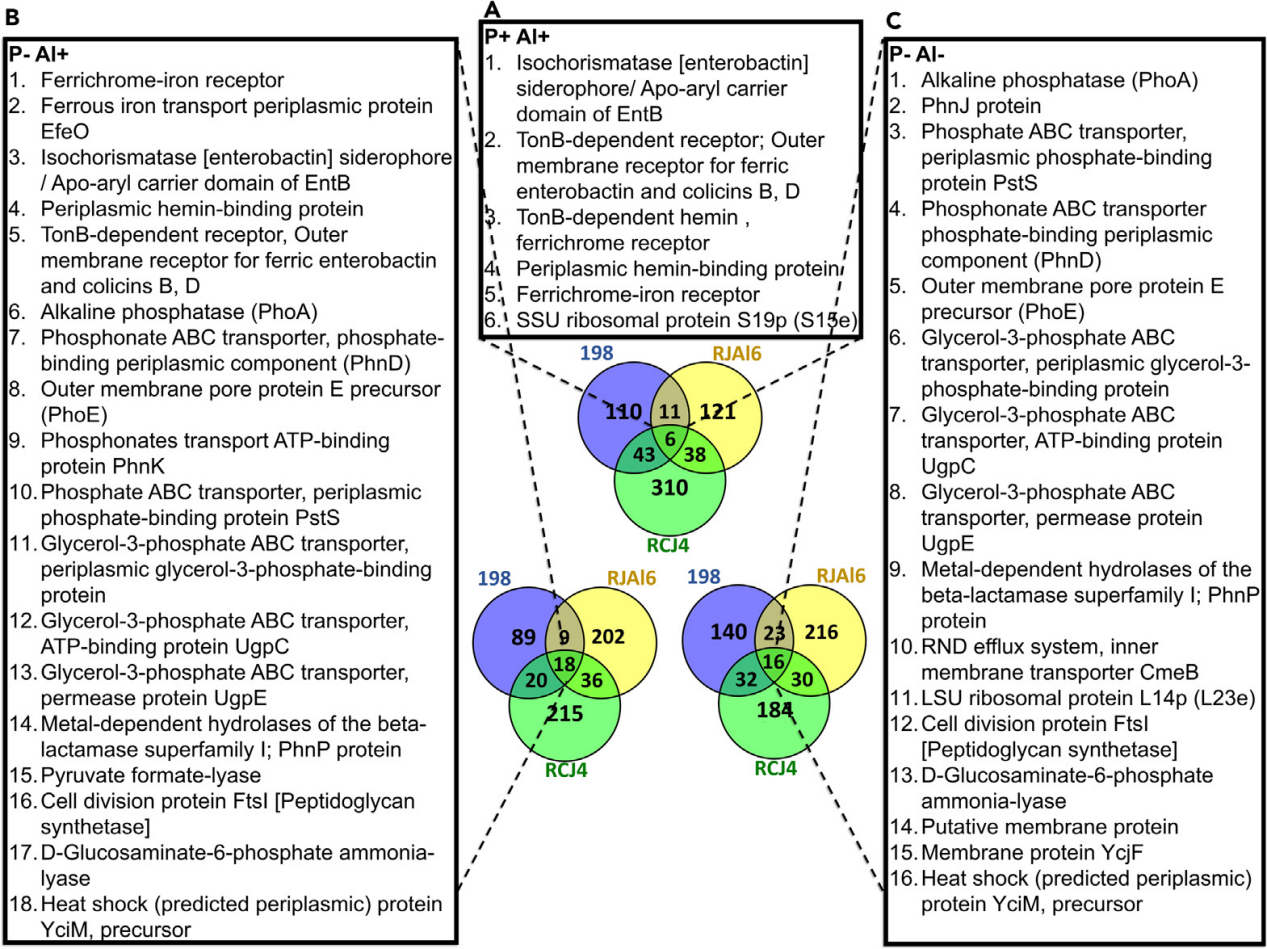
Proteins found to be differentially abundant in the three bacterial strains (A–C) Venn diagram of the proteins that were found crosswise being differentially abundant in the three bacterial strains evaluated (*Enterobacter* sp. 198, *Enterobacte*r sp. RJAL6 and *Klebsiella* sp. RCJ4) when subjected to (A) high Al (P + Al+), (B) P– deficiency and high Al (P– Al+) and (C) P deficiency (P– Al–) as compared with the control (P + Al–).

The presence of Al in the medium resulted in significant changes in the expression patterns of proteins classified into virtually each SEED category (Table 1). However, “iron acquisition and metabolism” was proportionally the most Al-affected category, with 29%, 35%, and 52% of proteins classified into this category being significantly (p ≤ 0.05, log_2_ fold change ≥ 2) induced in the strains 198, RJAl6 and RCJ4, respectively. The Venn diagram (Figure 4) shows that six proteins were differentially expressed across the three strains when challenged with Al, five of which are coincidentally involved in Fe acquisition and metabolism: (1) Periplasmic hemin-binding protein (HmuT); (2) Isochorismatase [enterobactin] siderophore/Apo–aryl carrier domain of EntB (EntB); along with three TonB-dependent receptors, these are: (3) Ferrichrome-iron receptor (FhuA); (4) TonB-dependent hemin, ferrichrome receptor (HemR); and (5) TonB-dependent receptor outer membrane receptor for ferric enterobactin and colicins B, D (FepA).

**Figure 5:**
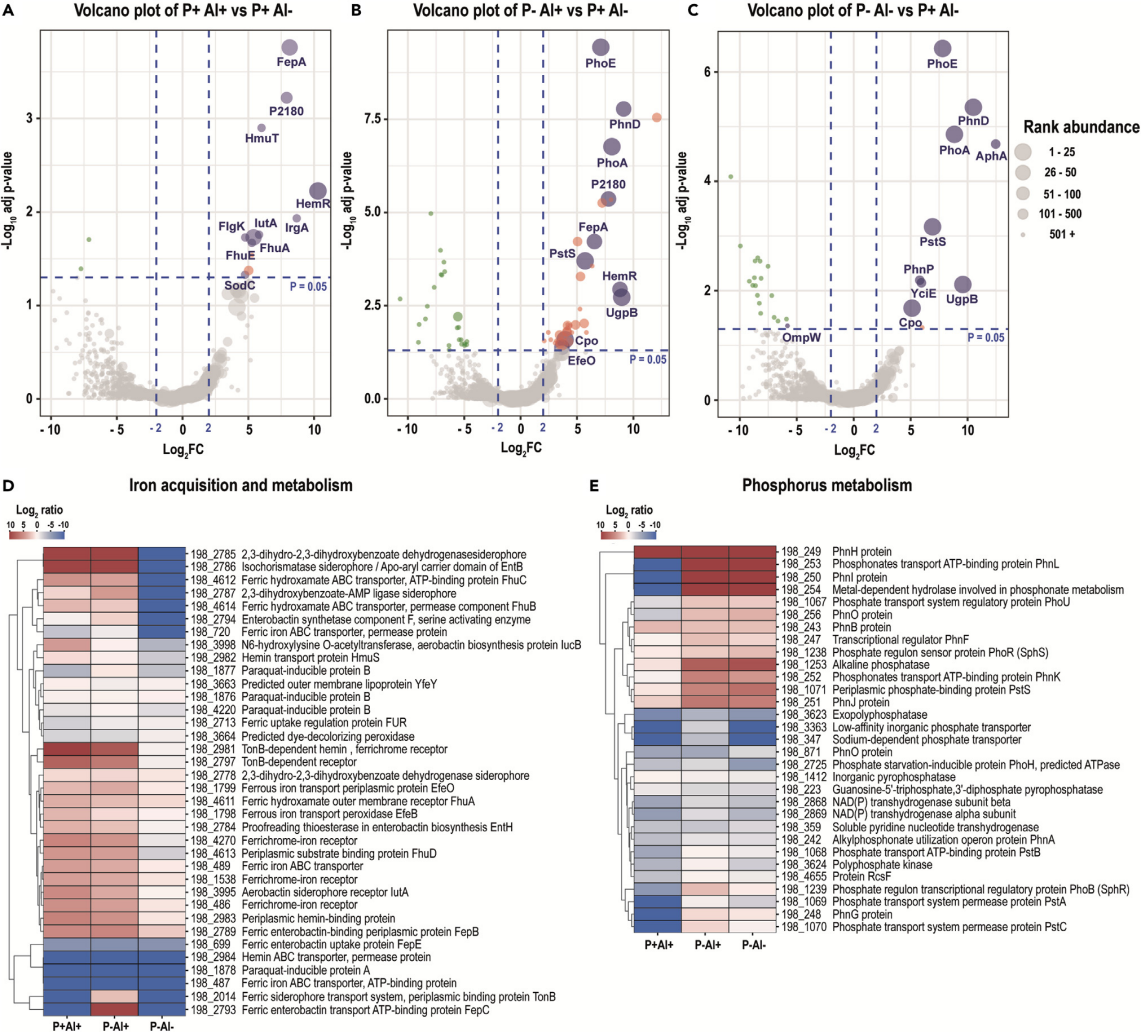
Volcano plots and heatmaps of the main categories of proteins affected by Al stress and P deficiency in the bacterial strain *Enterobacter* sp. 198 Volcano plots depicting detected proteins in the bacterial strain *Enterobacter* sp. 198 when comparing (A) high Al (P+ Al+), (B) P deficiency and high Al (P– Al+) and (C) P deficiency (P– Al–) versus the control (P+ Al–). Volcano plots illustrate the statistical p value (y-axis) versus the relative abundance ratio, Log_2_ fold change (Log_2_FC, x-axis). The horizontal hatched line indicates the p = 0.05 threshold, while the left and right vertical hatched lines indicate log_2_ fold change of −2 and +2, respectively. Significantly downregulated (p value ≤ 0.05, log_2_ fold change ≤ 2) proteins in the stress treatment with respect to the control are shown in green (left), and those that are significantly upregulated (p value ≤ 0.05, log_2_ fold change ≥ 2) are in red (right). The rank abundance of each protein is denoted by dot size. Labeled dots (purple) represent the top ten most abundant proteins detected in each proteome. To improve readability of the volcano plots those proteins that were detected in some of the treatments but were absent in the control, or vice versa (presence/absence), were excluded from the graphic representation. Heatmap of proteins categorized into (D) “iron acquisition and metabolism category” and (E) “Phosphorus metabolism” based on RAST and the SEED subsystem analyses of the strain *Enterobacter* sp. 198.

**Figure 6:**
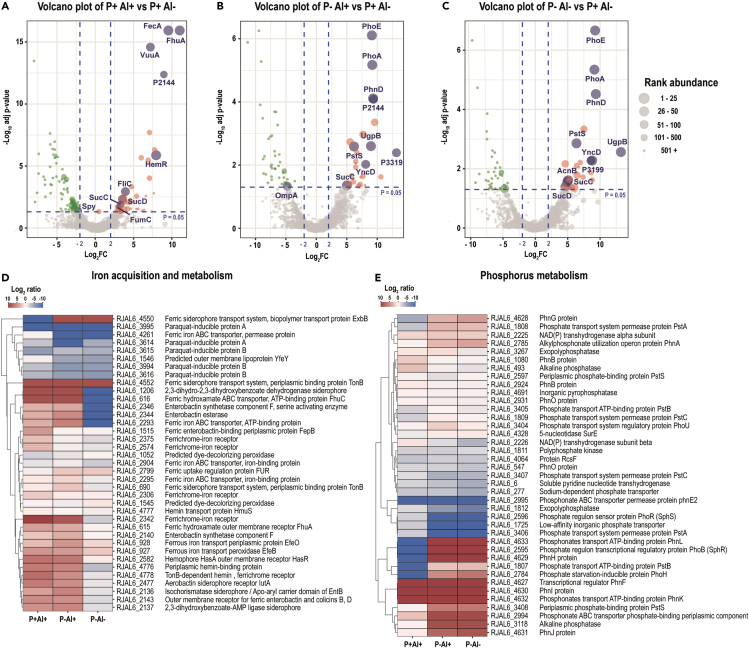
Volcano plots and heatmaps of the main categories of proteins affected by Al stress and P deficiency in the bacterial strain *Enterobacter* sp. RJAL6 Volcano plots depicting detected proteins in the bacterial strain *Enterobacter* sp. RJAL6 when comparing (A) high Al (P+ Al+), (B) P deficiency and high Al (P– Al+) and (C) P deficiency (P– Al–) versus the control (P+ Al–). Volcano plots illustrate the statistical p value (y-axis) versus the relative abundance ratio, Log_2_ fold change (Log_2_FC, x-axis). The horizontal hatched line indicates the p = 0.05 threshold, while the left and right vertical hatched lines indicate log_2_ fold change of −2 and +2, respectively. Significantly downregulated (p value ≤ 0.05, log_2_ fold change ≤ 2) proteins in the stress treatment respect to the control are shown in green (left), and those that are significantly upregulated (p value ≤ 0.05, log_2_ fold change ≥ 2) are in red (right). The rank abundance of each protein is denoted by dot size. Labeled dots (purple) represent the top ten most abundant proteins detected in each proteome. To improve readability of the volcano plots those proteins that were detected in some of the treatments but were absent in the control, or vice versa (presence/absence), were excluded from the graphic representation. Heatmap of proteins categorized into (D) “iron acquisition and metabolism category” and (E) “Phosphorus metabolism” based on RAST and the SEED subsystem analyses of the strain *Enterobacter* sp. RJAL6.

**Figure 7:**
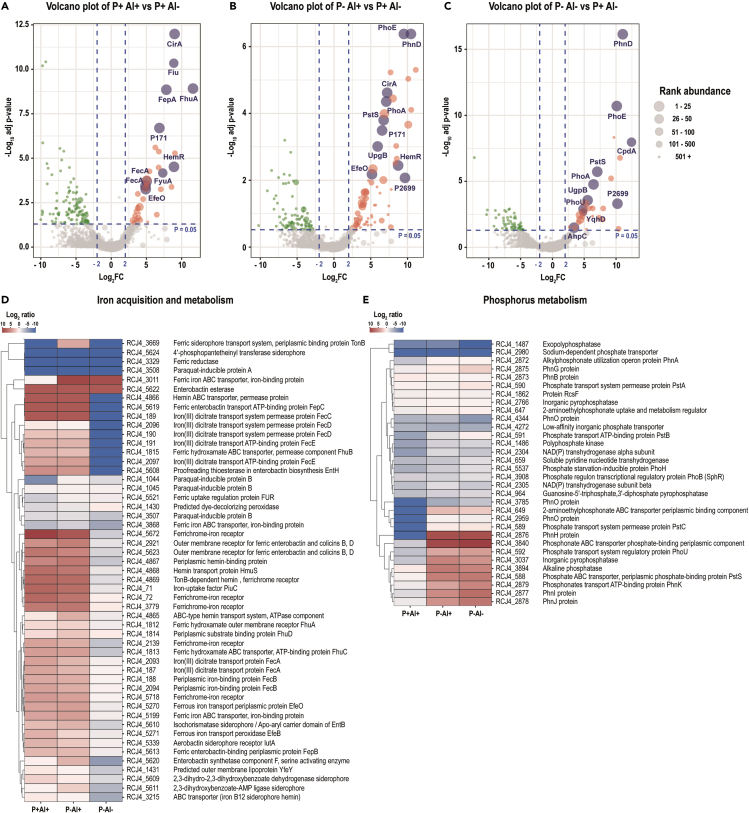
Volcano plots and heatmaps of the main categories of proteins affected by Al stress and P deficiency in the bacterial strain *Klebsiella* sp. RCJ4 Volcano plots depicting detected proteins in the bacterial strain *Klebsiella* sp. RCJ4 when comparing (A) High Al (P+ Al+), (B) P deficiency and high Al (P– Al+) and (C) P deficiency (P– Al–) versus the control (P+ Al–). Volcano plots illustrate the statistical p value (y-axis) versus the relative abundance ratio, Log_2_ fold change (Log_2_FC, x-axis). The horizontal hatched line indicates the p = 0.05 threshold, while the left and right vertical hatched lines indicate log_2_ fold change of −2 and +2, respectively. Significantly downregulated (p value ≤ 0.05, log_2_ fold change ≤ 2) proteins in the stress treatment with respect to the control are shown in green (left), and those that are significantly upregulated (p value ≤ 0.05, log_2_ fold change ≥ 2) are in red (right). The rank abundance of each protein is denoted by dot size. Labeled dots (purple) represent the top ten proteins detected in each proteome. To improve readability of the volcano plots those proteins that were detected in some of the treatments but were absent in the control, or vice versa (presence/absence), were excluded from the graphic representation. Heatmap of proteins categorized into (D) “iron acquisition and metabolism category” and (E) “Phosphorus Metabolism” based on RAST and the SEED subsystem analyses of the strain *Klebsiella* sp. RCJ4.

Among the top ten most abundant proteins (according to normalized abundance levels), seven, three, and nine are proteins involved in Fe acquisition and metabolism in the strains 198, RJAl6 and RCJ4, respectively (Figures 5A, 6A, and 7A; Table S6). These proteins include various TonB-dependent receptor proteins such as, FhuA, colicin I (CirA), ferric vulnibactin (VuuA), catecholate siderophore (Fiu), ferrienterochelin and colicins (IrgA), yersiniabactin (FyuA) and FepA, and transporters such as iron III dicitrate transport protein (FecA) and ferrous iron transport periplasmic protein (EfeO) (Figures 4A, 5A, and 6A, Table S6). It should be noted that several of these proteins are classified as “membrane transport” or “uncategorized” proteins according to SEED criteria, although their roles in Fe metabolism and acquisition are obvious. These findings are consistent with the results obtained from the PCA-SOM analysis, where it is shown that those nodes enriched with proteins classified in the “iron acquisition and metabolism” category, i.e., node 7, node 2, and node 12 in the bacterial strains 198 (Figure 1), RJAl6 (Figure 2) and RCJ4 (Figure 3), were significantly overrepresented when bacteria were grown in presence of Al. Therefore, Fe-related proteins were consistently found to be increased under Al stress as illustrated in the heat maps (Figures 5D, 6D, and 7D). In contrast, the iron-storage proteins such as ferritin-like protein, bacterioferritin and non-specific DNA-binding protein Dps (iron-binding ferritin-like antioxidant protein/ferroxidase) were downregulated by Al (Tables S7–S9). This tendency in response to high Al doses was also observed in various Fe-dependent enzymes, such as fumarate reductase, several peptides from the NADH-ubiquinone oxidoreductase (respiratory Complex I), superoxide dismutase [Fe] and fumarate hydratase class I in all strains, cytochromes (d and o) ubiquinol oxidase, succinate dehydrogenase iron-sulfur protein (except in 198), and NADH dehydrogenase (except in RJAl6). It is important to note that most of the downregulated Fe-containing proteins have a fundamental role in bacterial respiration and carbohydrate metabolism i.e., in energy generating metabolic pathways. Additional proteins involved in respiration and in carbohydrate metabolism such as glycerol dehydrogenase, citrate lyase, 3-ketoacyl-CoA thiolase/Acetyl-CoA acetyltransferase and citrate succinate antiporter, (except in RCJ4), maltodextrin phosphorylase, Na(+)–translocating NADH-quinone reductase, ribose-phosphate pyrophosphokinase, and glycogen phosphorylase (except in RCJ4), among other strains specific proteins were significantly downregulated under when a high Al level is present (Tables S7–S9). Moreover, several proteins involved in respiration and in carbohydrate metabolism such as succinyl-CoA ligase, fumarate hydratase class II, aldehyde dehydrogenase, acetyl-CoA synthetase, 3-ketoacyl-CoA thiolase, and acetyl-CoA acetyltransferase were upregulated under higher Al, but only in the strain RJAL6. Therefore, it is not surprising that the abundance of proteins from “respiration” category in the strain RJAl6 (node 11, Figure 2) and in the strain RCJ4 (node 6, Figure 3) was decreased in response to Al as compared with the control (P + Al–). Finally, the abundance of proteins grouped into the category “carbohydrates” and “nucleosides and nucleotides” in the strain RJAL6 (node 5, Figure 2) and in “stress response” in the strain 198 (node 8, Figure 1) were overrepresented in presence of Al.

### Bacterial response to P deficiency and high Al (P– Al+)

The impact of Al along with P deficiency on the proteome of the three phosphobacteria was evaluated in the modified media P–Al+. A total of 1967, 1783, and 2017 (Table S3) proteins were identified in the strain 198, RJAl6 and RCJ4. When the bacteria were subjected to P deficiency and high Al and, a total of 143, 287, and 304 proteins showed significant differences (p value ≤ 0.05, log_2_ fold-change ≥ 2) in abundance compared with the control (P + Al–) of which 61, 63, and 201 were common for both treatments with high Al doses (P + Al+ and P–Al+) in the strains 198, RJAl6 and RCJ4, respectively (Tables S3 and S7–S9). A higher number of the significantly abundant proteins (65.8%–71.0%) were lower under P–Al+ as compared with the control in the three bacterial strains, most of which were below the detection limit (Table S4).

Proteins classified within the SEED categories “phosphorus metabolism” and “iron acquisition and metabolism” had the greatest proportion of representatives showing significant differences in abundance under P deficiency and high Al with respect to the control (Table 1, Table S4). Among the proteins with significant differences, eighteen were identified transversally across the three bacterial strains (Figure 4). Fourteen of these proteins showed higher abundance levels in the P–Al+ treatment as compared with the control. Most of proteins were classified into “iron acquisition and metabolism” (five), “phosphorus metabolism” (five), “membrane transport” (three), and “carbohydrates” category (four). It is important to clarify that several of these proteins can be classified into more than one category. Only four proteins: (1) Pyruvate formate-lyase, (2) Cell division protein FtsI [Peptidoglycan synthetase], (3) D-Glucosaminate-6-phosphate ammonia-lyase and, (4) Heat shock (predicted periplasmic) protein YciM, precursor showed significantly greater abundance in the control as compared with the P–Al+ treatment.

The five proteins from the “iron acquisition and metabolism” category (i.e., isochorismatase, periplasmic hemin-binding protein, ferrichrome-iron receptor, ferrous iron transport periplasmic protein EfeO, and tonB-dependent receptor/outer membrane receptor for ferric enterobactin and colicins B, D) transversally upregulated in the P–Al+ were also induced in the P + Al+ treatment (log_2_ fold change between 2.5 and 10.0). The proteins transversally increased from the “carbohydrates” category corresponded to three glycerol-3-phosphate ABC transporters. The five increased proteins from “phosphorus metabolism” category were alkaline phosphatase (PhoA), phosphonate ABC transporter phosphate-binding periplasmic component (PhnD), Outer membrane pore protein E precursor (PhoE), phosphonate transport ATP-binding protein (PhnK), and phosphate ABC transporter-periplasmic phosphate-binding protein (PstS). Protein PstS had the highest abundance among the significantly upregulated proteins in the three bacterial strains subjected to P deficiency and Al stress (Figures 5B, 6B, 7B; Tables S6–S9).

As expected, several proteins from the “phosphorus metabolism” category subject to Pho regulon control, such as PhoA, PhnD, and PhoE were among the top ten most abundant proteins under P deficiency and high Al in the three bacterial strains. Some additional proteins not categorized within “phosphorus metabolism” but also involved in P acquisition, such as periplasmic glycerol-3-phosphate-binding protein (UgpB, in the three strains) and outer membrane non-heme chloroperoxidase (Cpo, in the strain 198) were also found among the top ten most abundant proteins in the P–Al+ treatments (Figures 5B, 6B, and 7B). Some proteins from “iron acquisition and metabolism” category, such as protein EfeO and hemR (in the strains 198 and RCJ4), FepA (in the strain 198), YncD (in the strain RJAl6), and CirA (in the strain RCJ4) were also found among the proteins with highest levels of abundance among the differentially expressed proteins (Figures 5B, 6B, and 7B). Additionally, outer membrane protein A precursor (OmpA) and succinyl-CoA ligase [ADP-forming] beta chain (SucC) were found among the most abundant proteins in the strain RJAl6. Curiously, OmpA was significantly downregulated in P–Al+ as compared with the control.

The PCA-SOM analysis revealed that in the treatment P–Al+, as in the other high-dose Al treatment (P + Al+), the nodes enriched with proteins from “iron acquisition and metabolism” category, i.e., node 7, node 2, and node 12 for the bacterial strains 198 (Figure 1), RJAl6 (Figure 2), and RCJ4 (Figure 3), respectively, were also significantly overrepresented. Moreover, the nodes enriched with proteins from “phosphorus metabolism” category i.e., node 11, node 9, and node 2 for strain 198, RJAl6 and RCJ4, respectively, were overrepresented when the bacteria were grown under P deficiency and a high Al level. Heatmaps depict the “iron acquisition and metabolism” (Figures 5D, 6D, and 7D) and “phosphorus metabolism” (Figures 5E, 6E, and 7E) categories of the bacterial strains when subjected to a high Al dose, P deficiency and the combination of both factors as compared with the control (P + Al–). Similar than in the P-sufficient treatment, iron-storage proteins were also downregulated in P-deficient treatment in response to Al, with the exception of the non-specific DNA-binding protein Dps (Tables S7–S9).

As demonstrated the PCA-SOM analysis, several additional SEED categories of proteins were also altered under P–Al+ conditions, however, the responses were not consistent in the three bacterial strains. Thus, the nodes enriched with proteins from the categories, “stress response” in the strain 198 (node 8, Figure 1) as well as “nucleosides and nucleotides” and “carbohydrates” in the strain RJAl6 (node 5, Figure 2) were also overrepresented in the treatment P–Al+. In contrast, the nodes enriched with proteins from the categories “amino acids and derivatives” in the strain 198 (node 2, Figure 1), and “respiration” in the strains RJAl6 (node 11, Figure 2) and RCJ4 (node 6, Figure 3) were overrepresented in the control.

Lastly, it is important to highlight that several proteins were exclusively affected by Al in the P-sufficient treatment (P + Al+), demonstrating a significant contrast with the P-deficient treatment P–Al+ and the control P + Al–. These include proteins involved in copper homeostasis, such as multicopper oxidase, copper resistance protein B and copper resistance protein CopC in the strains 198 and RCJ4 and the copper-binding protein PcoE in the strain RCJ4, all of which were upregulated in P + Al+ as comparing with P– Al+. Whereas a probable copper-binding protein in the strain RCJ4 and the copper homeostasis protein CutE in the strains 198 and RCJ4 were downregulated in P + Al+ (Tables S7–S9).

### P deficiency (P– Al–)

When the bacterial strains 198, RJAl6 and RCJ4 were subjected to only P deficiency (P–Al–) a total of 1854, 1762, and 2970 proteins were identified (Table S3). Of these proteins, 214, 317, and 269 (Tables S3 and S7–S9) showed significant differences in abundance compared to the control (p value ≤ 0.05, log_2_ fold change ≥ 2). A total of 79, 253, and 204 proteins showed differences in both P deficiency treatments (Tables S3 and S7–S9). Among the proteins with significant differences, 7.1%, 26.9%, and 16.4% had higher abundance levels under P deficiency as compared with the control (Table S4). Like the other treatments, P deficiency resulted in significant changes in proteins classified within nearly every SEED category. As expected, the highest proportion of proteins significantly affected by P deficiency were those from the “phosphorus metabolism” category, most of which were induced. Among the proteins with significant differences, sixteen were crosswise identified in the three bacterial strains (Figure 4). Five of these proteins, PhoA, PhnJ, PhoE, and two phosphate ABC transporter (protein PstS and PhnD) are classified into the “phosphorus metabolism” category and four additional proteins are also related with P metabolism and transport, but not classified within the P metabolism category. These proteins are: PhnP protein, a metal-dependent phosphodiesterase of the beta-lactamase superfamily I involved in phosphonate degradation, from the category “virulence, disease and defense,” and three subunits of the ABC transport system for the uptake of glycerol-3-phosphate (UgpB protein, ATP-binding protein UgpC and permease protein UgpE), from the “carbohydrates” category.

As in the other P deficiency treatment (P–Al+), among the proteins with significant differences, PstS was found to have the highest abundance among the three bacterial strains (Tables S4–S6, Figures 5C, 6C and 7C). Other proteins also involved in the metabolism of P, such as PhoA, PhoE, PhnD, and UgpB were also found among the top ten most abundant proteins in the three bacterial strains when subjected to P deficiency (Figures 5C, 6C, and 7C, Table S6). Acid phosphatase (AphA) and PhnP protein in the strain 198, the alpha and beta chain of Succinyl-CoA ligase (SucC and SucD) and Aconitate hydratase 2 (AcnB) in the strain RJAl6, as well as, the phosphate transport system regulatory (protein PhoU) and alkyl hydroperoxide reductase protein C (AhpC) in strain RCJ4 were also found among the top 10 proteins (Figures 5C, 6C, and 7C; Table S6). Tables S7–S9 shows all the differentially abundant proteins with respect to the control in the strains 198, RJAl6 and RCJ4, respectively.

The PCA-SOM analysis (Figures 1, 2, and 3) revealed that the nodes enriched with proteins from “phosphorus metabolism” category i.e., node 11, node 9, and node 2 in strains 198, RJAl6 and RCJ4, respectively, were the only ones that were transversely overrepresented in the three bacterial strains grown under P deficiency (P–Al–). However, nodes enriched with other categories of proteins were also significantly affected under P deficiency, although not consistently in the three bacterial strains. Thus, the nodes enriched with proteins from the categories, “stress response” in strain 198 (node 8, Figure 1), as well as “nucleosides and nucleotides” and “carbohydrates” in strain RJAl6 (node 5, Figure 2) were also overrepresented in the treatment P–Al+. The nodes enriched with proteins from the categories “protein metabolism” in the strain 198 (node 1, Figure 1) and in the strain RCJ4 (node 11, Figure 3) and “respiration” in the strains RJAl6 (node 11, Figure 2) were overrepresented in the control. It is also important to note that a number of proteins from the “protein metabolism” category (33, 10, and 23 in the strains 198, RCJ4 and RJAl6, respectively), were downregulated under P-deficient conditions. The proteins include various LSU and SSU ribosomal proteins (Tables S7–S9). Proteins involved in iron metabolism in general did not show significant differences with respect to the control when subject to P deficiency. A comprehensive and schematic summary of our main findings can be found in the graphical abstract.

## Discussion

Plant-associated bacteria have been extensively investigated as potential plant growth improvers in acid soils, due to their intrinsic capacity to enhance soil P availability and to reduce the adverse effects of environmental stresses on plants (e.g., Al toxicity).^31^^,^^41^ However, how Al-tolerant bacteria naturally inhabiting acidic soils respond to high levels of Al under both P-sufficient and deficient conditions is poorly known, especially at the molecular level. Therefore, we evaluated by label-free quantitative proteomics the response of three Al-tolerant phosphobacteria strains to P deficiency, high Al levels and the combination of both factors. The results revealed that although each bacterial strain has a unique behavior, there are some common response patterns. The most striking and relevant finding is the preponderant role that Al plays in the modification of Fe homeostasis, and consequently in the central metabolism of the bacteria. Although several previous studies have shown that Al can interfere with cellular Fe metabolism, especially through the replacement of Al by Fe, the molecular mechanisms detailing how biological systems deal with the apparent Fe deficiency and subsequent toxicity have not been fully delineated.^4^^,^^13^^,^^42^

Here, we determined that proteins implicated both in the capture and transport of Fe, including precursors of siderophores, receptors, and transporters involved in the uptake of Fe-siderophore and other Fe-organic ligands were notoriously upregulated in the three Al-tolerant bacterial strains when grown under high doses of Al. This response could be a consequence of two main survival strategies, with the production of a wide spectrum of siderophores fulfilling a pivotal role. One strategy revolves around safeguarding themselves from the harmful effects of Al by impeding its entry into their intracellular space. This defensive mechanism works in conjunction with alterations in the patterns of extracellular exudation of low-molecular-weight organic acids that aid in sequestering Al.^30^^,^^37^ These mechanisms, previously described as plant growth-promoting (PGP) traits, could potentially cf. benefits to host the host plants as well. The second survival strategy focuses on optimizing the acquisition and maintenance of Fe. In our study we observed overproduction of proteins involved in the synthesis of siderophores such as isochorismatase and enterobactin synthetase when the bacteria were grown in the presence of Al. Enterobactin has been described as the siderophore with the greatest known affinity for Fe.^43^ It is well known that under Fe-limiting conditions most aerobic bacteria, and other microorganisms, produce siderophores with a high affinity for Fe^3+^.^42^^,^^44^ Bacteria capture these Fe-siderophore compounds and take them up through specific high-affinity systems.^45^^,^^46^ However, Al^3+^, having chemical similarity to Fe^3+^, has been shown to form complexes with siderophores, although with lower affinity.^10^^,^^42^^,^^44^^,^^47^ Overproduction of siderophores in response to high doses of Al has already been documented.^10^^,^^37^^,^^42^^,^^44^^,^^48^ In this context, Braud et al., (2010)^44^ suggested that pyoverdine and pyochelin, two siderophores produced by *Pseudomonas aeruginosa* reduces toxic metal accumulation in bacteria and increases metal tolerance, including Al. The authors indicated that both siderophores were able to sequester Al, as well others metals, in the extracellular medium, decreasing metal diffusion into the bacteria. Similar findings were reported by Mora et al. (2017),^37^ who demonstrated the formation and accumulation of extracellular Al-siderophore (hydroxamate type) complexes when bacteria, including the strains *Enterobacter* sp. RJAL6 and *Klebsiella* sp. RCJ4, were grown in a media supplemented with Al, but without Fe. The autofluorescence of these extracellular Al-siderophore complexes was observed by confocal scanning microscopy. Nevertheless, Al when present in excess can still be transported into the cell by both passive uptake and siderophore-mediated uptake.^10^ An augmented availability of siderophores would thus not only act preventing the entry of Al to the cell but also would be an additional mechanism for metal uptake considering that apparently the cell does not discriminate between the Fe-siderophore and Al-siderophore complexes.^10^ Therefore, under an eventual Al excess, the bacteria would not be able to exclude all metal entering the cell generating a consequent pressure on the cellular metabolism.^49^

In addition to the overproduction of proteins involved in siderophore synthesis, we demonstrated that receptors and transporters of Fe-chelator complexes involved in Fe acquisition were also increased under high Al. Such receptors, identified as outer membrane proteins are usually overexpressed in response to Fe starvation.^46^^,^^50^ A concomitant downregulation in proteins involved in intracellular Fe storage, Fe-containing proteins and redox-active metalloenzymes was also observed in response to high Al doses. These findings support the second proposed bacterial strategy to deal with Al excess, which is aimed at optimizing the acquisition and homeostasis of Fe since an underlying effect of elevated levels of Al is the development of intracellular Fe deficiency.^4^ Although Fe is a highly abundant element in soils, it is not easily incorporated since in aerobic environments it is readily oxidized from the ferrous (Fe^2+^) to ferric (Fe^3+^) state, which can form insoluble hydroxides.^51^ Excess Al may thus further impair Fe availability. In a system where Al:Fe ratio is high a cell might not perceive the presence of Fe.^52^ For example, *Pseudomonas fluorescens* can fluoresce under Fe starvation^50^ and also when exposed to high levels of Al,^37^^,^^52^ evidencing that Al can impair Fe acquisition. Al did not stimulate siderophore production in Fe-replete cultures,^10^ suggesting that the response here reported was a result of Fe limitation caused by high Al rather than a direct effect of Al. Since Fe is implicated in many vital biological processes, such as energy generating metabolic pathways, both in the oxidative phosphorylation and the TCA cycle, detoxification of ROS, DNA synthesis and repair, among others, its intracellular depletion has a direct effect on the overall cellular metabolism.^46^^,^^53^^,^^54^ As several central enzymes involved in carbohydrate metabolism and oxidative phosphorylation are iron-sulfur (Fe-S) or hem-containing proteins, Fe limitation leads to an malfunction of energy metabolism.^4^^,^^46^^,^^53^^,^^55^ Fumarate hydratase, succinate dehydrogenase, and aconitate hydratase are key iron-containing enzymes participating in the TCA cycle. In line with previous studies,^4^^,^^11^^,^^34^^,^^52^ we determined that the levels of these iron-containing enzymes, with the exception of aconitate hydratase, were significantly downregulated by Al at the same time as the levels of the iron-independent fumarate hydratase class II were increased. Our findings agree with Chenier et al., (2008), who demonstrated that although the activity of the enzyme aconitate hydratase was significantly decreased, its levels were not affected in the Al-challenged *P. fluorescens* as evidenced by 2D SDS-PAGE and immunoblotting assays.^56^ In contrast, the authors determined an abrupt decrease in the levels of succinate dehydrogenase and fumarate hydratase class I, while fumarate hydratase class II increased following Al exposure.^56^ Therefore, the lack of fumarate hydratase class I, which catalyzes the stereospecific interconversion of fumarate to L-malate, is compensated by rising the expression of the Fe-independent enzyme fumarate hydratase class II, thus making possible to keep the TCA cycle active. A concomitant shift of the metabolism to utilize the glyoxylate shunt, evidenced as increased activity of isocitrate lyase and isocitrate dehydrogenase (NADP), after a decreased aconitase activity have also been previously described in response to Al.^4^^,^^57^ Our results did not show an activation of this metabolic bypass, possibly because aconitase levels were not significantly affected by Al under the experimental conditions of this study. Several Fe-dependent proteins from electron transport complexes, such as NADH-ubiquinone oxidoreductase, cytochromes d, and cytochromes o ubiquinol oxidase were also downregulated by Al. These transmembrane complexes are involved in the proton translocation that provides the electrochemical gradient indispensable for the aerobic ATP synthesis by ATPase.^58^ Therefore, the process of ATP production via oxidative phosphorylation is perturbed, although not completely inhibited, in Al-challenged bacteria. To compensate for the drop in energy production, bacteria overproduce the enzyme succinyl-CoA ligase, which catalyzes the reversible reaction of succinyl-CoA to succinate and generates ATP (or GTP) via substrate-level phosphorylation. This phenomenon had previously been described in *P. fluorescens* challenged with Al.^58^ The increase in succinyl-CoA ligase activity generates increased levels of succinate, which can be secreted along with others low-molecular-weight organic acids and helps in the extracellular sequestration of Al.^30^

The redox homeostasis of the Al-challenged cells might also be affected by the respiratory malfunction with the potential of accumulating NAD(P)H while decreasing their reducing power.^51^ Therefore, bacteria must rearrange their metabolism to regenerate NAD(P)^+^ in order to maintain the redox balance. An increase in the activity and expression of H_2_O-forming NADH oxidase along with a decrease in NADH-producing enzymes of the TCA cycle, such as isocitrate dehydrogenase and 2-oxoglutarate dehydrogenase, have been proposed as strategies to balance redox homeostasis under high intracellular-Al.^4^^,^^56^^,^^58^^,^^59^ However, these responses were not evidenced under the conditions of our study. Based in our results, we propose that several flavoproteins, such as NAD(P)H dehydrogenase (quinone), (EC 1.6.5.2), and FMN oxidoreductase (NADPH dehydrogenase; EC:1.6.99.1), which utilizes flavin cofactors instead of Fe to catalyze the oxidation of NADH and the zinc enzyme quinone oxidoreductase (EC 1.6.5.5) would have a preponderant role in the regeneration of NAD(P)^+^ since their levels were maintained and even generally increased (although not significantly) in Al-challenged bacteria. Additional studies are necessary to corroborate this hypothesis. In addition, Al is a pro-oxidant element, leading to increased ROS production.^4^ Superoxide dismutase represents one of the main cellular defense mechanisms against oxidative stress.^60^ However, similar to other Fe-dependent proteins, the levels of the Fe-dependent superoxide dismutase isoform were significantly decreased in Al-challenged bacteria, which to a certain extent would deepen the cellular oxidative stress. To cope with this oxidizing environment, the bacteria responded mainly increasing the levels of the Mn-dependent superoxide isoenzyme, and to a lesser extent, the Cu-Zn isoenzyme. Although we detected an overproduction of proteins involved in Fe uptake under Al+ conditions, the underlying mechanisms of this response in these Al-tolerant strains are not entirely elucidated at present. Secondly, it is unclear whether these strains exhibit a superior ability compared to more Al-sensitive bacteria to selectively sequester Fe or exclude Al-chelator complexes. These aspects require in-depth investigation to shed light on their significance. To our knowledge, this is the first proteomic study showing that the response of bacteria challenged with a high dose of Al resembles the response of a bacterium subject to Fe deficiency,^61^ in other words, optimizing Fe acquisition and homeostasis. It is important to note that our study was conducted with low Fe concentrations, aiming to minimize the Fenton reaction, which, under high Fe concentrations, can lead to an increased generation of ROS. Consequently, further studies incorporating varying Fe concentrations should be undertaken to validate and reinforce our current findings.

We also investigated the effect of P limitation in the three phosphobacteria strains, which showed an increase of enzymes, transporters, and receptors involved in P acquisition and metabolism, including the proteins PhoA, PhnD, PhoE, PhnK, and PstS. All these proteins are members of the Pho regulon, a suite of genes that code for proteins required for scavenging inorganic P or for the use of alternative P sources.^62^ Indeed, the PstS protein, the periplasmic substrate-binding protein of the high-affinity inorganic P transporter, PstSCAB,^39^ showed the highest levels of induction in the three bacterial strains. Strong overproduction of the PstS protein is a well-known phenomenon in bacteria exposed to P starvation.^20^^,^^40^^,^^63^^,^^64^ Our findings confirm that a main strategy of plant-associated bacteria to acquire, and to compete for P, comprises the production of enzymes responsible for the mineralization of P-containing organic (organophosphorus or organophosphates) compounds, such as alkaline phosphatase, as well as transporters involved in the uptake of organophosphorus compounds such as phosphomonoesters, phosphonates, and glycerol-3-phosphate. The phosphomonoesters that are hydrolyzed by phosphatases are generally the dominant fraction of organophosphorus compounds.^65^ Therefore, overexpression of phosphatase is a typical bacterial response to P-starvation. We previously reported increased intracellular and extracellular alkaline phosphatase activity in the strains *Enterobacter* sp. RJAL6 and *Klebsiella* sp. RCJ4 in response to P deficiency.^30^ When P is in limited supply several bacterial species are also able to metabolize phosphonate compounds, which are ancient biogenic molecules that contain the chemically stable C–P bond.^66^ The usage of these compounds as P source require specialized enzymatic machinery to break this stable bond.^67^ The main route of phosphonate metabolism involves the broad-specificity C–P lyase multi-enzyme complex that comprises fourteen proteins encoded by the *phn* operon.^66^ The enzyme complex utilizes a wide range of compounds as substrate including alkyl, amino-alkyl, and aryl phosphonates and releases inorganic phosphate,^67^ Here, we showed incremented levels of seven of these proteins, PhnA, PhnH, PhnI, PhnJ, PhnK, PhnL, and PhnP, under P-deficiency, indicating that this complex plays a key role under P deficiency. Here, we also showed an increased level of the proteins UgpB, UgpC, and UgpE. These proteins are part of the Pho regulon-dependent Ugp system, responsible for the transmembrane transport of glycerol-3-phosphate. This system consists of the periplasmic glycerol-3-phosphate-binding UgpB protein, the integral membrane proteins UgpE and UgpA, and the UgpC permease.^68^ The use of glycerol-3-phosphate and others organophosphorus compounds as an alternative P source for bacteria under P-starvation is an already known phenomenon.^20^^,^^69^ However, our knowledge about the bacterial enzymatic rearrangement in response to nutritional stress continues to be partial, as modern molecular studies have been gradually demonstrating.^20^^,^^40^^,^^70^ This is especially evident when more than one stressor is present, which is the norm in many soils and environments. In this context, in our study several proteins were also observed to be differentially expressed exclusively between both high Al treatments, namely between P + Al+ and P–Al+ conditions. Among these, several proteins involved in copper homeostasis stand out. It is possible that sufficient P may enhance bacterial antioxidant defense systems, upregulating copper homeostasis proteins for ROS detoxification. In contrast, P deficiency could render bacteria more susceptible to ROS-induced damage, exacerbating the combined impact of Al toxicity and P deficiency in the absence of adequate copper homeostasis. However, the reason for the differential expression of these proteins under P contrasting conditions when bacteria are Al-challenged should be further studied. Despite differential expression of some proteins, our results thus demonstrated that bacterial responses in general were rather influenced by Al and P separately, than the mixture of both factors.

### Conclusions

Here, using a proteomic approach, we showed the response of three Al-tolerant bacterial strains to two major stressors typically found in acidic soils, high Al content, and low P availability. Although each strain had particular responses, several common patterns of metabolic rearrangement were evident in all three strains. Among these, the ability of the three bacterial strains to overproduce proteins responsible for obtaining organophosphate compounds when subject to P deficiency is noteworthy. These results support the ecological and agronomic importance of plant-associated bacteria in the P mineralization from soil organic forms. We also demonstrated the main role that Al excess plays in the modification of Fe homeostasis, and therefore, in the central metabolism of soil bacteria. Since bacterial proteins involved in Fe mobilization and uptake were highly overexpressed, our study indicates that Al could be a key factor involved in the modulation of the Fe cycle in the soil-plant continuum. Nevertheless, because this is the first proteomic study of Al toxicity and its interaction with P deficiency in acidic soil-adapted bacteria, further studies using complementary omics approaches, and also comparing with more Al-sensitive bacteria are still necessary to confirm this hypothesis. The knowledge generated in this study is crucial to understanding bacterial behavior in acidic soils, which in turn will be important for the generation of efficient biofertilizers for crops grown in acidic soils.

### Limitations of the study

In the present investigation, we delved into the intricate interplay between aluminum (Al) excess and phosphorus (P) deficiency, elucidating their impact on the proteomic responses of three distinct aluminum-tolerant and phosphate-solubilizing bacterial strains, all of which were isolated from acidic soils. However, it is paramount to acknowledge a key limitation within our study framework. Specifically, we acknowledge that our exploration remained confined to bacteria exhibiting Al tolerance and P solubilization traits. Regrettably, we did not extend our analysis to encompass bacterial strains that lack the capacity to tolerate Al or enhance P availability. This exclusion, due to the scope of our investigation, does highlight a potential avenue for future research to broaden the comprehension of microbial responses across a wider spectrum of traits. Our findings also evidence an apparent iron (Fe) deficiency triggered by the presence of excessive Al. In hindsight, our study’s comprehensiveness could have been augmented through an examination of escalating Fe dosages within the cultivation medium. By juxtaposing these different Fe concentrations, a more nuanced understanding of the intricate nutrient interactions could have been attained, potentially furnishing deeper insights into the observed proteomic responses. Moreover, the bacterial isolates used in this study were sourced exclusively from acidic soil environments. The intricate interplay between environmental factors and bacterial behavior suggests that broader generalizations necessitate a more diverse sampling of bacterial strains across varying contexts.

## STAR★Methods

### Key resources table

**Table:** 

REAGENT or RESOURCE	SOURCE	IDENTIFIER
**Bacterial and virus strains**		

*Enterobacter* sp. 198	Barra et al.^71^	KR066646
*Enterobacter* sp. RJAL6	Mora et al.^37^	KU697295
*Klebsiella* sp. RCJ4	Mora et al.^37^	KU697296

**Chemicals, peptides, and recombinant proteins**		

Proteomics grade trypsin	Sigma-Aldrich	Cat. #T6567
C18 PepMapTM trap column	Thermo Scientific	Cat. #ES-903
75–mm id x 50–cm C18 Pep–Map column	Thermo Scientific	Cat. #160454

**Critical commercial assays**		

Dneasy UltraClean Microbial kit	Qiagen	Cat. #12224-50
Nextera XT DNA Library Prep Kit	Illumina	Cat. #FC-131-1096
BugBuster Master Mix protein extraction reagent	Merck Millipore	Cat. #US171456-3
Protein Assay Dye Reagent Concentrate	Bio-Rad	Cat. #BR-5000006

**Deposited data**		

Raw data and analyzed genome: Strain *Enterobacter* sp. 198	This article	GenBank BioProject: PRJNA575621
Raw data and analyzed genome: Strain *Enterobacter* sp. RJAL6	This article	GenBank BioProject: PRJNA575626
Raw data and analyzed genome: Strain *Klebsiella* sp. RCJ4	This article	GenBank BioProject: PRJNA575624
Proteomics dataset to *ProteomeXchange* Consortium via the PRIDE^72^: Strain *Enterobacter* sp. 198	This article	PXD041866
Proteomics dataset to ProteomeXchange Consortium via the PRIDE^72^: Strain *Enterobacter* sp. RJAL6	This article	PXD041868
Proteomics dataset to *ProteomeXchange* Consortium via the PRIDE^72^: *Klebsiella* sp. RCJ4	This article	PXD041867

**Software and algorithms**		

FastQC	Andrews^73^	https://github.com/s-andrews/FastQC
Trimmomatic v0.3.2	Bolger et al.^74^	https://github.com/usadellab/Trimmomatic
Abyss v2.3.7	Jackman et al.^75^	https://github.com/bcgsc/abyss
RAST server	Brettin et al.^76^	http://rast.theseed.org
BLAST (Basic Local Alignment Search Tool)	M adden^77^	https://blast.ncbi.nlm.nih.gov/Blast.cgi
*Prodigal* v2.6.3	Hyatt et al.^78^	https://github.com/hyattpd/prodigal
*Statmod 1.5.0*	Giner and Smyth^79^	https://cran.r-project.org/web/packages/statmod/
*proteiNorm*	Graw et al.^80^	https://github.com/ByrumLab/proteiNorm
*Kohonen 3.0.3*	Wehrens and Kruisselbrink)^81^	https://cran.r-project.org/web/packages/kohonen
*Goseq 1.52.0*	Young et al.^82^	https://bioconductor.org/packages/goseq/
*pheatmap 1.0.12*	(Kolde)^83^	https://cran.r-project.org/web/packages/pheatmap
*ggplot2 3.4.0*	Wickham^84^	https://cran.r-project.org/web/packages/ggplot2/
*R 4.1.0*	R Core Team	https://www.r-project.org
MS data acquisition sofware *Xcalibur* 4.1	Thermo Fisher Scientific, San Jose, CA	https://www.thermofisher.com/order/catalog/product/fr/fr/OPTON-30965
MS data processing software *Proteome Discoverer* 2.2	Thermo Fisher Scientific, San Jose, CA	OPTON-31099

**Other**		

Ultimate 3000 nano LC system	Thermo Fisher Scientific, San Jose, CA	https://www.thermofisher.com/order/catalog/product/fr/fr/ULTIM3000RSLCNANO
Electrospray Orbitrap Fusion™ Lumos™ Tribrid™ Mass Spectrometer	Thermo Fisher Scientific, San Jose, CA	Cat #IQLAAEGAAPFADBMBHQ

### Resource availability

#### Lead contact

Further information and requests for resources and reagents should be directed to and will be fulfilled by the lead contact, Dr Patricio Javier Barra (patricio.barra@ufrontera.cl).

#### Materials availability

This study did not generate new unique reagents.

### Experimental model and subject details

#### Bacterial strains

In this study, we focused on three Al-tolerant phosphobacteria strains, which were derived from previous research conducted by our study group.^30^^,^^37^^,^^71^^,^^85^ These strains include *Enterobacter* sp. 198 (KR066646),^71^ *Enterobacter* sp. RJAL6 (KU697295) and *Klebsiella* sp. RCJ4 (KU697296).^37^^,^^86^ The strain 198 was isolated from the rhizosphere of avocado (32°50′58″ S-71°00′08″ W; soil pH of 5.95; 0.17% Al saturation)^71^ and the strains RJAl6 and RCJ4 from the rhizosphere of Ryegrass (39°06′12″ S-72°37′42″ W; soil pH of 5.3; 24% Al saturation).^37^ Comprehensive characterization of these strains was carried out, as documented in Barra et al. (2016, 2017, 2018) and Mora et al. (2017). The decision to include these particular strains in our current study was primarily due to their remarkable ability to tolerate high concentrations of aluminum (10 mM). Moreover, these strains exhibited proficiency in solubilizing inorganic P and mineralizing organic P forms through the release of organic acids and the enzymatic activity of intracellular and extracellular phosphatases.^37^^,^^86^ Additionally, the selected strains were found to produce siderophores and auxins,^37^^,^^71^ utilize ACC as the sole N source,^37^^,^^71^ and promote plant growth, such as wheat,^71^ ryegrass^31^^,^^37^ and/or avocado^85^ subjected to environmental stresses or P deficiency.

To assess the Al tolerance of our selected strains, we employed the minimum inhibitory concentration (MIC) method, following the methodology as described by Mora et al. (2017). As for strain 198, which had not been subjected to such assessment, we conducted the determination of its organic acid secretion in the supernatant of bacterial cultures (Figure S1), employing the same methodology employed for strains RCJ4 and RJAL6, as described in Barra et al., (2018). The analysis of extracellular phosphatase activity was carried out using the methodology described in Barra et al., 2018, utilizing the supernatant of bacterial cultures. Additionally, the production of auxins and the utilization of ACC as the sole N source, was determined as described in Barra et al. 2016. Furthermore, for the evaluation of siderophore production, we followed the methodology provided in Mora et al., (2017).

### Methods details

#### Sequencing of phosphobacteria genomes

The three selected bacterial strains were grown overnight in 10 mL of LB broth at 30°C in a rotatory shaker with constant agitation (120 rpm). Bacterial cells were collected by centrifugation at 3,000 × g for 4 min. Total genomic DNA was extracted using the Dneasy UltraClean Microbial kit (Qiagen, Inc.) according to the manufacturer’s instructions. The quantity and quality of the extracted DNA was determined using a MultiskanTM GO Microplate Spectrophotometer (Thermo Fisher Scientific Inc.). The whole genomes of the phosphobacteria were *de novo* sequenced using Illumina HiSeq 4,000 platform at Macrogen Inc. (Seoul, Republic of Korea) with 100 bp paired–end reads. The DNA libraries were prepared using a Nextera XT DNA Library Prep Kit (Illumina, San Diego, CA, USA) according to the manufacturer’s protocol.

The quality of the reads was checked and trimmed (Phred quality score >15) with Trimmomatic version 0.3.2^74^ and FastQC tools.^73^ A *de novo* assembly was carried out using A5–MiSeq pipeline.^87^ In this pipeline, Basic Ribosomal RNA Predictor (available at https://github.com/tseemann/barrnap) and HMMER^88^ tools were used to predict the location of ribosomal RNA genes and protein sequence homologs in genomes, respectively. In relation with the taxonomic assignment, identified 16S rRNA genes were compared with those deposited in the Ribosomal Database Project (https://rdp.cme.msu.edu/) and by the Basic Local Alignment Search Tool (BLAST; https://blast.ncbi.nlm.nih.gov/Blast.cgi) from the National Center for Biotechnology Information (NCBI). All sequences were deposited in the GenBank BioProject under the accession numbers: PRJNA575621 (Strain 198), PRJNA575624 (Strain RCJ4) and PRJNA575626 (Strain RJAl6).

#### Protein extraction from bacteria under high Al and P deficiency treatments

##### Culture media

The standard mineral culture medium (MCM) described by Barra et al., (2018) was initially used to grow the three selected phosphobacteria strains. The MCM contained (L^−1^): 1.0 g KCI, 1.0 g NH_4_Cl, 0.01 g CaCl_2_ × 2 H_2_O, 0.87 g K_2_SO_4_, 0.2 g MgSO_4_ × 7 H_2_O and 1.0 mg FeSO_4_ × 7 H_2_O. The only P source used was KH_2_PO_4_ at a concentration of 400 μM. Trace elements included in this medium are described in Barra et al., (2018). The MCM was buffered at pH 5.4 with citrate buffer. This choice of pH was made to align our experimental conditions with previous literature^9^^,^^30^ and importantly, to ensure that the growth of the bacterial strains remained unaffected, as this pH closely approximated the native soil pH of the bacterial strains. The citrate was also employed as the sole C source in the culture medium at a concentration of 4.0 g L^−1^. Four adapted MCM media were used to analyze the effect of P deficiency, high Al levels and of both factors combined on the phosphobacteria proteome. These media were previously formulated by Barra et al., (2018) in order to achieve: (i) P sufficiency without Al (P + Al–; control), (ii) P sufficiency and high Al (P + Al+), (iii) P deficiency without Al (P– Al–), and (iv) P deficiency and high Al (P– Al+). The sufficiency (P+) and deficiency (P–) of P in the media was obtained by adding KH_2_PO_4_ at final concentration of 1.40 and 0.05 mM, respectively, as described by Lidbury et al., (2016). Whereas, to achieve a high Al level, but avoiding Al precipitation, AlCl_3_ × 6 H_2_O at a concentration of 10 mM was complexed to the citrate prior to sterilization as described by Appanna and St Pierre, (1994).^89^ Thus, Al–citrate was used as the sole C source in the Al+ treatments. The three selected bacterial strains were cultured in modified MCM at 30°C and 150 rpm. Growth was monitored by measuring the change in optical density at 600 nm (OD600) using a MultiskanTM GO Microplate Spectrophotometer (Thermo Fisher Scientific Inc.). OD600 measurements were taken automatically every 15 min for a period of 24 h.

#### Growth conditions of phosphobacteria and total protein extraction

The three selected phosphobacteria strains were initially incubated overnight in 10 mL of the standard MCM at 30°C on a rotary shaker at 120 rpm. Then, the phosphobacteria were centrifuged at 3,200 rpm for 5 min and the pellets were repeatedly washed with sterile 0.85% NaCl solution, resuspended and finally diluted to an optical density of 0.1 at 600 nm (OD_600_). 50–μL aliquots of each of the phosphobacteria suspensions were inoculated in triplicate into 10 mL of the four modified MCM media. All cultures of phosphobacteria were grown at 30°C on a rotary shaker at 120 rpm. Phosphobacteria cultures were harvested during the late exponential phase of growth. The final density and harvest time of the bacterial culture depended on the treatment and bacterial strain as described by Barra et al., (2018).^30^ For this, 2 mL of each culture were centrifuged at 14,000 × *g* for 10 min in a pre–weighed Eppendorf tube. The collected bacterial cells were lysed with BugBuster Master Mix protein extraction reagent (Novagen, Madison, USA) following the manufacturer’s recommendations. While the primary objective of using BugBuster Master Mix was to achieve maximum recovery of soluble proteins, it is important to note that this reagent also allowed us to recover insoluble proteins. The crude bacterial cell extracts obtained were treated with protease inhibitor cocktail (Sigma–Aldrich, Inc; St. Louis, USA) according to the manufacturer’s protocol. Total protein concentration was determined colorimetrically by the Bradford method.

#### Proteomic analysis

##### Sample preparation and protein digestion

Protein samples were solubilized in Laemlli buffer and 5 μg aliquots were loaded onto SDS–PAGE gel to concentrate and clean the samples. Separation was stopped once proteins had entered the resolving gel. After colloidal blue staining, bands were cut out from the SDS–PAGE gel and subsequently cut in 1 mm × 1 mm gel pieces. Gel pieces were destained in 25 mM ammonium bicarbonate 50% acetonitrile (ACN), rinsed twice in ultrapure water and shrunk in ACN for 10 min. After ACN removal, gel pieces were dried at room temperature, covered with trypsin solution (10 ng μl^−1^ in 50 mM NH_4_HCO_3_), rehydrated at 4°C for 10 min, and finally incubated overnight at 37°C. Gel pieces (spots) were then incubated for 15 min in 50 μL NH_4_HCO_3_ 50 mM at room temperature on a rotary shaker. The supernatants were collected, and 50 μL of an H_2_O/ACN/HCOOH (47.5:47.5:5) extraction solution was added onto the gel slices for 15 min. The extraction step was repeated twice. Supernatants from all three individual incubations were pooled and concentrated in a vacuum centrifuge to a final volume of 100 μL. Digests were finally acidified by addition of 2.4 μL of formic acid and stored at −20°C.

##### nLC–MS/MS analysis and label–free quantitative data analysis

The peptide mixtures were analyzed on an Ultimate 3000 nano LC system (Dionex, Amsterdam, The Netherlands) coupled to an Electrospray Orbitrap Fusion Lumos Tribrid Mass Spectrometer (Thermo Fisher Scientific, San Jose, CA). Ten microliters of peptide digests were loaded onto a 300–μm–inner diameter x 5–mm C18 PepMapTM trap column (LC Packings) at a flow rate of 10 μL min^−1^. The peptides were eluted from the trap column onto an analytical 75–mm id x 50–cm C18 Pep–Map column (LC Packings) with a 4–40% linear gradient of solvent B in 45 min (solvent A was 0.1% formic acid and solvent B was 0.1% formic acid in 80% ACN). The separation flow rate was set at 300 nL min^−1^. The mass spectrometer operated in positive ion mode at a 1.8–kV needle voltage. Data were acquired using Xcalibur 4.1 software in a data–dependent mode. MS scans (m/z 375–1,500) were recorded at a resolution of R = 120,000 (@ m/z 200) and an AGC target of 4 × 10^5^ ions collected within 50 ms. Dynamic exclusion was set to 60 s and top speed fragmentation in HCD mode was performed over a 3 s cycle. MS/MS scans with a target value of 3 × 10^3^ ions were collected in the ion trap with a maximum fill time of 300 ms. Additionally, only +2 to +7 charged ions were selected for fragmentation. Other settings were as follows: no sheath nor auxiliary gas flow, heated capillary temperature, 275°C; normalized HCD collision energy of 30% and an isolation width of 1.6 m/z. Monoisotopic precursor selection was set to Peptide and an intensity threshold was set to 5 × 10^3^.

### Quantification and statistical analysis

#### Bacterial growth

The statistical significance of the growth curves was assessed using the *CompareGrowthCurves* function from the R package statmod (version 1.5.0), with 10,000 permutations. To account for multiple testing, the p values were adjusted using Holm’s method, which is implemented in the *compareGrowthCurves* function.

#### Database search and results processing

Data were searched by SEQUEST through Proteome Discoverer 2.2 (Thermo Fisher Scientific Inc.) against a custom database for each bacteria strain, built from the protein prediction based on *Prodigal* v2.6.3. Spectra from peptides higher than 5,000 Da or lower than 350 Da were rejected. The search parameters were as follows: mass accuracy of th monoisotopic peptide precursor and peptide fragments was set to 10 ppm and 0.6 Da respectively. Only b– and y–ions were considered for mass calculation. Oxidation of methionines (+16 Da) and protein N–terminal acetylation (+42Da) were considered as variable modifications and carbamidomethylation of cysteines (+57 Da) as fixed modification. Two missed trypsin cleavages were allowed. Peptide validation was performed using the Percolator algorithm^90^ and only ‘high confidence’ peptides were retained corresponding to a 1% false positive rate at peptide level. Peaks were detected and integrated using the Minora algorithm embedded in Proteome Discoverer. Proteins were quantified based on unique peptide intensities. Normalization was performed based on total protein amount. Protein ratios were calculated as the median of all possible pairwise peptide ratios. A t–test was calculated based on the background population of peptides or proteins. Quantitative data were considered for proteins quantified by a minimum of 2 peptides, fold changes above 2, and a statistical p value lower than 0.05. Proteins were functionally categorized based on RAST and the SEED subsystem analysis (Subsystem and FIGfams Technology). The mass spectrometry proteomics data have been deposited to the *ProteomeXchange* Consortium via the PRIDE^72^ partner repository with the dataset identifier: PXD041866 (*Enterobacter* sp. 198), PXD041867 (*Klebsiella* sp. RCJ4) and PXD041868 (*Enterobacter* sp. RJAL6).

The measured intensity values obtained from the proteomic experiments were normalized by variance stabilization normalization (VSN) and imputed with the K–Nearest Neighbor method (KNN) using proteiNorm. To identify abundance patterns across samples, the proteins were clustered using dimensionality reduction with a Principal Component Analysis (PCA, Figure S2), followed by a 2 × 6 Self–Organizing Map (SOM) using one hundred training iterations, as implemented in the kohonen package in R.^91^ The PCA–SOM output was visualized as a pie chart for codebook vectors to obtain the counts and mean distance of the proteins assigned to each node. The abundance patterns of each node were visualized in a boxplot to associate their accumulation with any treatment. Each node was analyzed for enrichment of SEED categories with a false discovery rate (FDR) of 0.05 using GOSeq^82^ and SEED categories as terms. The protein abundance patterns related to Fe Metabolism and Phosphorus Metabolism were also visualized as a heatmap using the log_2_ fold change and with a volcano plot including both log_2_ fold change and the –log_10_ p value. The graphs were constructed using pheatmap and ggplot2 libraries in R statistical environment.^84^

## Data Availability

• The mass spectrometry proteomics data have been deposited to the ProteomeXchange Consortium via the PRIDE^72^ partner repository partner repository with the dataset identifiers PXD041866 (*Enterobacter* sp. 198), PXD041868 (*Enterobacter* sp. RJAL6) and PXD041867 (*Klebsiella* sp. RCJ4). • Raw sequencing data and draft genome assemblies have been deposited to NCBI BioProject with the dataset identifiers PRJNA575621 (*Enterobacter* sp. 198), PRJNA575626 (*Enterobacter* sp. RJAL6) and PRJNA575624 (*Klebsiella* sp. RCJ4).

## Supplemental information

Document S1. Figures S1 and S2

Document S2. Tables S1–S9 Table S1. Plant growth-promoting traits of selected aluminum-tolerant bacteria used in this Study. Table S2. Statistical analysis of the growth of selected bacterial strains: *Enterobacter* sp. 198, *Klebsiella* sp. RCJ4, *Enterobacter* sp. RJAL6 in a modified mineral culture medium with contrasting P and Al concentrations. The growth of the bacterial strains was compared under different conditions, including P+ (1.4 mM KH_2_PO_4_), P– (0.05 mM KH_2_PO_4_), Al+ (10 mM), and Al– (without Al added). The treatments were compared by using *CompareGrowthCurves* function from the 'statmod' package in R. Table S3. Number of differentially abundant proteins in each of the treatments. Table S4. Number of differentially abundant proteins (present, absent, down-regulated, and up-regulated) grouped according to the SEED subsystem in the strains *Enterobacter* sp. 198, *Enterobacter* sp. RJAl6 and *Klebsiella* sp. RCJ4 when subject to Al toxicity (P+ Al+), P- deficiency (P- Al-) and the combination of both stressors (P- Al+) versus unstressed control (P+ Al-). Table S5. Common and exclusive proteins found in the proteomes of the three analyzed bacterial strains, *Enterobacter sp*. 198, *Enterobacter* sp. RJAl6 and *Klebsiella* sp. RCJ4, independent of treatment. Table S6. Top ten most abundant proteins detected in each proteome of the strains *Enterobacter sp*. 198, *Enterobacter* sp. RJAl6 and *Klebsiella* sp. RCJ4 when subjected to high Al (P+ Al+), P- deficiency and high Al (P- Al+) and P-deficiency (P- Al-). Table S7. Differentially abundant proteins in the strain *Enterobacter* sp. 198 when subject to high Al (P+ Al+), P- deficiency and high Al (P- Al+) and P-deficiency (P- Al-) versus unstressed control (P+ Al-). The orange, yellow and blue colors show the proteins significantly abundant in P+Al+, P-Al+ and P-Al- as compared to the control, respectively. Table S8. Differentially abundant proteins in the strain *Enterobacter* sp. RJAl6 high Al (P+ Al+), P- deficiency and high Al (P- Al+) and P-deficiency (P- Al-) versus unstressed control (P+ Al-). The orange, yellow and blue colors show the proteins significantly abundant in P+Al+, P-Al+ and P-Al- as compared to the control, respectively. Table S9. Differentially abundant proteins in the strain *Klebsiella* sp. RCJ4 when subject to high Al (P+ Al+), P- deficiency and high Al (P- Al+) and P-deficiency (P- Al-) versus unstressed control (P+ Al-). The orange, yellow and blue colors show the proteins significantly abundant in P+Al+, P-Al+ and P-Al- as compared to the control, respectively.
