# Supplementary material for: Proteomic response to phosphorus deficiency and aluminum stress of three aluminum-tolerant phosphobacteria isolated from acidic soils
Source: iScience. 2023 Sep 14;26(10):107910. doi: 10.1016/j.isci.2023.107910 (PMC10543181; doi:10.1016/j.isci.2023.107910)

iScience, Volume *26*

Supplemental information

Proteomic response to phosphorus deficiency and aluminum stress of three aluminum -tolerant phosphobacteria isolated from acidic soils

Patricio Javier Barra, Paola Duran, Mabel Delgado, Sharon Viscardi, Stéphane Claverol, Giovanni Larama, Marc Dumont, and María de la Luz Mora

Supplemental figure titles and legends.

Figure S1. Organic acid secretion: A. Oxalic acid, B. Citric acid, C. Malic acid, by the strain *Enterobacter* sp. 198 grown in mineral culture media with contrasting P and Al concentrations, including P+ (1.4mM KH_2PO_4), P– (0.05mM KH_2PO_4), Al+ (10mM), and Al– (without Al added). Bars with different letters indicate significant differences ($P \leq 0.05$; Tukey's test) in organic acid secretion among treatments.

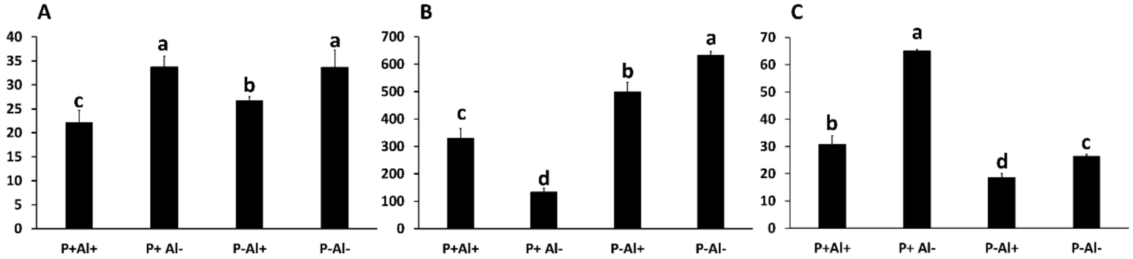

Figure S2. Principal component analysis (PCA) of each bacterial strain proteome, namely *Enterobacter* sp. 198 (a), *Enterobacter* sp. RJAL6 (b), and *Klebsiella* sp. RCJ4 (c), was conducted based on the normalized abundance and colored by their respective Self-Organizing Map (SOM) node.

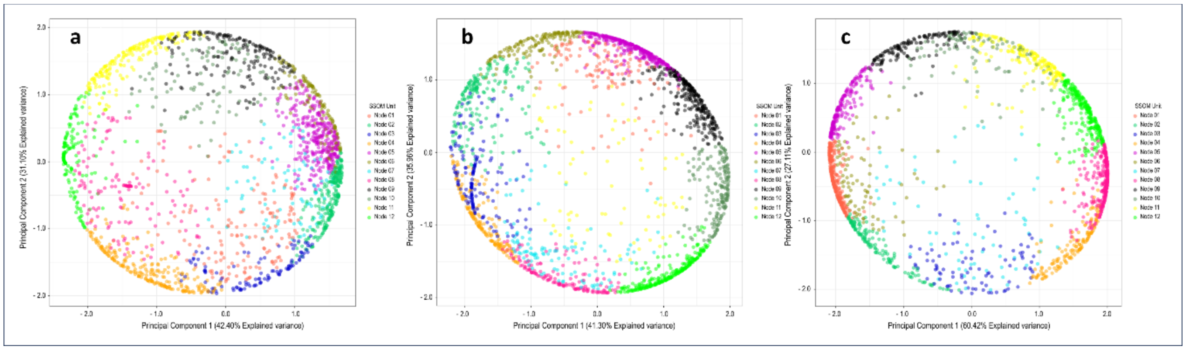

Supplement: Document S1. Figures S1 and S2 [file mmc1.pdf]
